# Direct evidence that late Neanderthal occupation precedes a technological shift in southwestern Italy

**DOI:** 10.1002/ajpa.24593

**Published:** 2022-07-20

**Authors:** Gregorio Oxilia, Eugenio Bortolini, Giulia Marciani, Jessica Cristina Menghi Sartorio, Antonino Vazzana, Matteo Bettuzzi, Daniele Panetta, Simona Arrighi, Federica Badino, Carla Figus, Federico Lugli, Matteo Romandini, Sara Silvestrini, Rita Sorrentino, Adriana Moroni, Carlo Donadio, Maria Pia Morigi, Viviane Slon, Marcello Piperno, Sahra Talamo, Carmine Collina, Stefano Benazzi

**Affiliations:** ^1^ Department of Cultural Heritage University of Bologna Ravenna Italy; ^2^ Department of Archaeology and Anthropology Institució Milà i Fontanals de Investigación en Humanidades, CSIC Barcelona Spain; ^3^ Department of Physical Sciences, Earth and Environment University of Siena, U. R. Preistoria e Antropologia Siena Italy; ^4^ Department of Enterprise Engineering “Mario Lucertini” University of Rome “Tor Vergata” Rome Italy; ^5^ Department of Physics and Astronomy “Augusto Righi” University of Bologna Bologna Italy; ^6^ CNR Institute of Clinical Physiology Pisa Italy; ^7^ CNR Institute of Environmental Geology and Geoengineering Milan Italy; ^8^ Department of Chemical and Geological Sciences University of Modena and Reggio Emilia Modena Italy; ^9^ Department of Earth Sciences, Environment and Resources University of Naples Federico II Naples Italy; ^10^ Max Planck Institute for Evolutionary Anthropology, Department of Evolutionary Genetics Leipzig Germany; ^11^ Tel Aviv University, Department of Anatomy and Anthropology and Department of Human Molecular Genetics and Biochemistry Sackler Faculty of Medicine Tel Aviv Israel; ^12^ Tel Aviv University, The Dan David Center for Human Evolution and Biohistory Research Tel Aviv Israel; ^13^ Museo Civico Archeologico Biagio Greco Mondragone Italy; ^14^ Department of Chemistry “G. Ciamician” University of Bologna Bologna Italy; ^15^ Department of Human Evolution Max Planck Institute for Evolutionary Anthropology Leipzig Germany

**Keywords:** deciduous human molars, Mediterranean Europe, Neanderthal, supervised learning algorithms, Uluzzian, virtual analysis

## Abstract

**Objectives:**

During the middle‐to‐upper Paleolithic transition (50,000 and 40,000 years ago), interaction between Neanderthals and *Homo sapiens* varied across Europe. In southern Italy, the association between *Homo sapiens* fossils and non‐Mousterian material culture, as well as the mode and tempo of Neanderthal demise, are still vividly debated. In this research, we focus on the study of two human teeth by using 3D geometric morphometric approaches for a reliable taxonomical attribution as well as obtaining new radiometric dates on the archeological sequence.

**Material and Methods:**

This work presents two lower deciduous molars uncovered at Roccia San Sebastiano (Mondragone‐Caserta, Italy), stratigraphically associated with Mousterian (RSS1) and Uluzzian (RSS2) artifacts. To obtain a probabilistic attribution of the two RSS teeth to each reference taxa group composed of Neanderthals and *Homo sapiens*, we performed and compared the performance of three supervised learning algorithms (flexible discriminant analysis, multiadaptive regression splines, and random forest) on both crown and cervical outlines obtained by virtual morphometric methods.

**Results:**

We show that RSS1, whose Mousterian context appears more recent than 44,800–44,230 cal BP, can be attributed to a Neanderthal, while RSS2, found in an Uluzzian context that we dated to 42,640–42,380 cal BP, is attributed to *Homo sapiens*.

**Discussion:**

This site yields the most recent direct evidence for a Neanderthal presence in southern Italy and confirms a later shift to upper Paleolithic technology in southwestern Italy compared to the earliest Uluzzian evidence at Grotta del Cavallo (Puglia, Italy).

## 1 INTRODUCTION

During the middle‐to‐upper Paleolithic transition (50,000 and 40,000 years ago), Europe witnessed a crucial population turnover characterized by the replacement of Neanderthals by *Homo sapiens* (Benazzi, Douka, et al., 2011; Hublin et al., 2020). In the same chronological interval, the archeological record offers evidence of a conspicuous, although spatially and temporally differentiated, shift in material culture and technology across the continent (Arrighi, Moroni, et al., 2020; Benazzi, Douka, et al., 2011; Hublin, 2015; Hublin et al., 2020; Marciani et al., 2020; Richter et al., 2009), which kindle still heated debates concerning the chronology of *Homo sapiens* migrations into Europe (Hublin et al., 2020), the impact of a possible interaction between *Homo sapiens* and Neanderthals, and the attribution of different techno‐complexes to different *Homo* species (Bar‐Yosef, 2007; Benazzi, Viola, et al., 2011; Hoffecker, 2009; Hublin, 2015; Marciani et al., 2020; Moroni et al., 2018; Slimak et al., 2022; Tostevin, 2003){FormattingCitation}.

**FIGURE 1:**
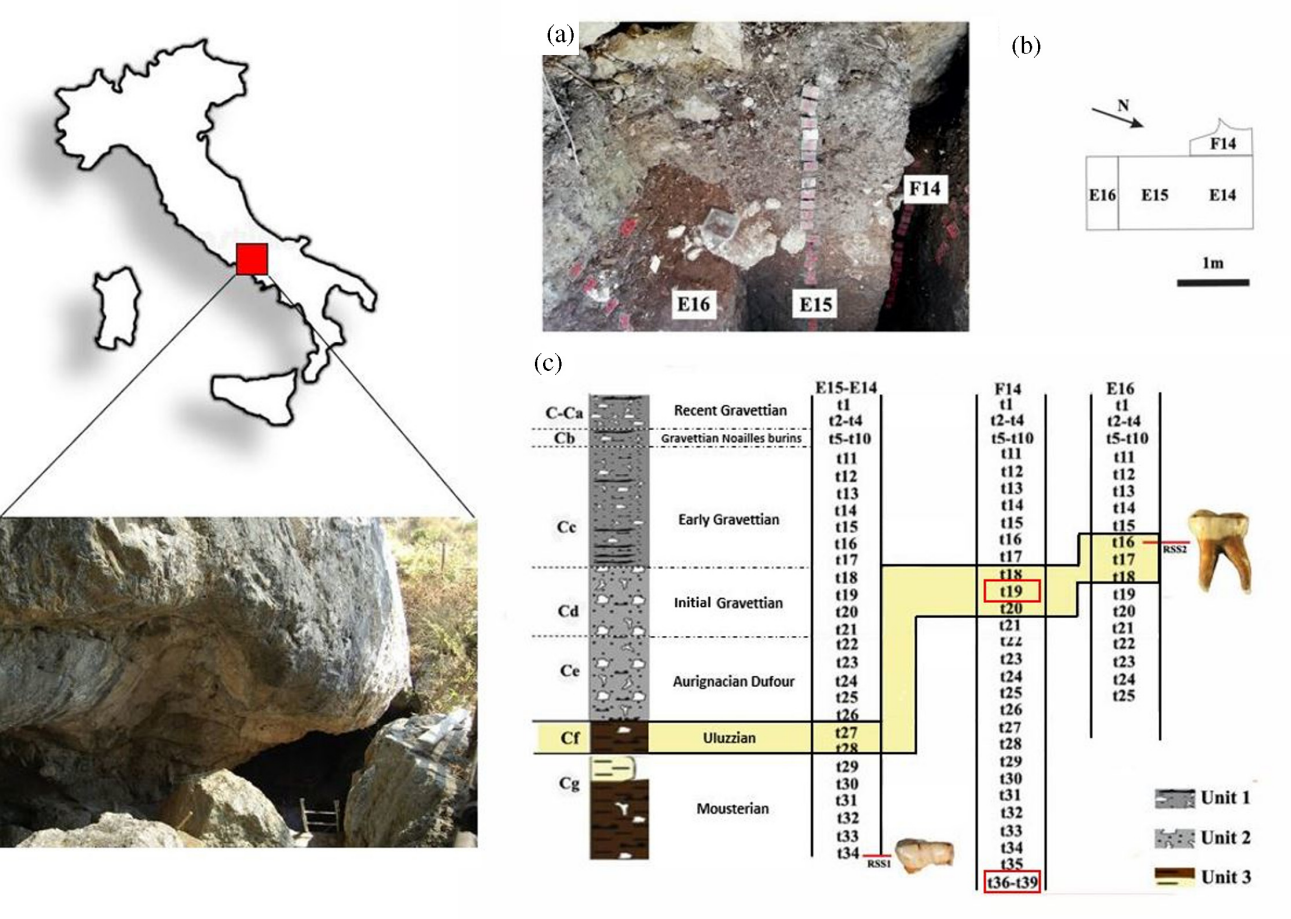
Stratigraphic sequence of Roccia san Sebastiano. (a) Detail of the excavation area (photos by C. Collina); (b) planimetry of the excavated areas; (c) stratigraphic sequence correlation, lithological column, cultural domain. Uluzzian levels (highlighted in yellow) and direct radiocarbon dating (red rectangle) have been highlighted

The Italian Peninsula plays a key role in the study of human evolution due to its geographical position (at the centre of the Mediterranean), broad environmental diversity (Badino et al., 2020) (See supplementary “Environmental setting”), and richness of archeological evidence (Benazzi, Viola, et al., 2011; Hoffecker, 2009; Marciani et al., 2020). However, only a handful of Neanderthals and *Homo sapiens* remains dated between ~50 and 40 ka cal BP have been found in Italy (Benazzi et al., 2014, 2015; Benazzi, Douka, et al., 2011; Fabbri et al., 2016; Moroni et al., 2018; Romandini et al., 2020; Zanchetta et al., 2018), thereby preventing a comprehensive overview on the relationship between these two species. The multi‐layered site of Roccia San Sebastiano (Mondragone‐Caserta, Southern Italy) (Figure 1) is of great interest because of (1) its location which confirms a dense prehistoric occupation on the Tyrrhenian side of Italy; (2) the richness and variety of its assemblages; and (3) the continuous settlement spanning from the middle‐to‐the upper Paleolithic (i.e., it attests several technocomplexes: Gravettian, Aurignacian Dufour, Uluzzian and Mousterian).

Here, we provide evidence of two left second lower deciduous molars (hereafter called RSS1 and RSS2) coming from the Roccia San Sebastiano that were discovered in the Late Mousterian (RSS1) and the Uluzzian (RSS2) deposits. We run state‐of‐the‐art attribution methods on two new human fossils and obtain new radiometric dates on the archeological sequence of RSS in order to (1) disentangle potential association with different human species and (2) ascertain the timing of middle‐to‐upper Paleolithic technological shift to compare to other areas in Italy (e.g., Apulia).

### 1.1 Archeological setting

Roccia San Sebastiano is a cavity of tectonic‐karstic origin opening in the Cretaceous limestone outcrops (paleogeographic units of Mt. Matese ‐ Mt. Maggiore) at the foot of the southern slopes of Monte Massico (Southern Apennines), in the Incaldana area (Mondragone‐Campania) (Belluomini et al., 2007; Collina et al., 2008; Collina, Benazzi, et al., 2020; Collina & Gallotti, 2007; Collina, Marciani, et al., 2020) (Figure 1). The cave (about 12 meters in length and 3 m in depth) is divided into two distinct parts: the outer portion named the rock shelter and the internal portion (whose dimensions have not yet been determined). The exploration of the chrono‐cultural sequence of the entire stratigraphic deposit is provided by three trenches (E14‐E15, F14, and E16) (see supplementary material).

The archeological sequence (Table S1) dug in trench E14–15 at Roccia San Sebastiano can be divided into three main units (labeled Units 1 to 3) (See supplementary “The site: stratigraphic sequence”). Unit 1 (sub‐units: C‐Ca ‐recent Gravettian, Cb‐Gravettian with Noailles burins, and Cc‐Early Gravettian). Unit 2 (sub‐units: Cd‐Initial Gravettian, and Ce‐Aurignacian Dufour) Unit 3 (sub‐units: Cf‐Uluzzian and Cg‐ Mousterian) (See supplementary “The site: lithic assemblages”).

## 2 MATERIAL AND METHODS

### 2.1 MicroCT scan and digital reconstruction

High‐resolution microCT images of the RSS1 Ldm2 were obtained with the XALT scanner (Panetta et al., 2012) at the Institute of Clinical Physiology CNR, Pisa (Italy). The tooth was scanned at 50 kVp, 2 mm Al filtration, 960 projection over 360°, 0.9 mAs/projection for a total scan time of 50 min per sample. The tomographic images were reconstructed using a parallelized version of a modified Feldkamp algorithm (Feldkamp et al., 1984) with included raw data filtration and correction procedures for various unwanted effects and artifacts. The final reconstructed volume consisted of an array of 950 × 950 × 770 isometric voxels, each with a side length of 13.8 μm.

MicroCT images of RSS2 Ldm2 were acquired at the Department of Physics and Astronomy of the University of Bologna. The tooth was scanned at 130 kVp, 0.1 mm Fe filtration, 900 projections over 360°, 1.32 mAs/projection for a total scan time of 279 min per sample. The tomographic images were reconstructed using the modified Feldkamp algorithm (Feldkamp et al., 1984) with embedded compensation for mechanical misalignments and raw data pre‐correction for beam‐heardening. The final reconstructed volume consisted of an array of 950 × 950 × 770 cubic voxels, each with a side length of 13.8 μm.

The microCT images of the original samples were virtually segmented using Avizo 9.2 software (Thermo Fisher Scientific, Waltham, Massachusetts, USA). The segmented enamel caps and virtually filled dentins were converted to meshes using the Geomagic Design X (3D Systems Software, Rock Hill, South Carolina, USA), a 3D metrology software.

### 2.2 Radiocarbon dating

Animal bones (Table 1) from which the collagen was extracted at the Department of Human Evolution, Max Planck Institute for Evolutionary Anthropology (MPI‐EVA) in Leipzig, Germany, were kindly provided by the Archaeological Museum Biagio Greco (Mondragone, Italy) through an official clearance. The extraction of collagen followed the pre‐treatment procedures in Talamo and Richards (2011) (MPI‐Code: R‐EVA) (see Supplementary Information for details).

**TABLE 1:** The results of AMS radiocarbon dating of the faunal remains discovered at the Roccia san Sebastiano cave.

MPI code	Quadrato	Strato	Sample code	Specie	Human modification	Collagen mass (mg)	Coll %	δ^13^C	δ^15^N	%C	%N	C:N	AMS code	^14^C age	1σ err	From Cal BP 68,32%	To Cal BP 68,3%	From Cal BP 95,4%	To Cal BP 95,4%
R‐EVA 3074	F14	T19	RSS2017‐F14‐T19‐17e	Cervus	X	5,2	0,85	−21,09	5,7	38,2	14,3	3,1	ETH‐99090.1.1	38,691	293	42,640	42,380	42,790	42,280
R‐EVA 3047	F14	T39	RSS2017‐F14‐T39‐13c	Indeterminato	X	5,3	0,95	−20,1	7	37,4	13,8	3,2	ETH‐99085.1.1	41,638	405	44,810	44,230	45,290	43,500

*Note*: The calibrated radiocarbon dates are provided using IntCal20 (Reimer et al., 2020) into the OxCal v. 4.4 program (Ramsey, 2009). δ^13^C values are reported relative to the vPDB standard, and δ^15^N values are reported relative to the AIR standard.

Abbreviations: MPI, Max Plank Institute; AMS, accelerator mass spectrometry, Cal BP, calibrated before present.

### 2.3 Morphological description and metric comparison

Morphological descriptions and morphometric analyses of the teeth were undertaken on the original specimens and also on the digital models. For the study of morphological traits of deciduous teeth, non‐metric dental traits were assessed following standards outlines adapted from Arizona State University Dental Anthropology System, (ASUDAS) (Turner et al., 1991) and deciduous dental morphology (Garralda et al., 2020; Hanihara, 1956), which was devised for *H. sapiens* dentition (Harvati et al., 2015; Riga et al., 2018). Occlusal wear stage (Oxilia et al., 2015, 2017, 2018; Oxilia, Bortolini, et al., 2021; Oxilia, Menghi Sartorio, et al., 2021) was scored based on Molnar (1971). Age at death was estimated by evaluating tooth formation and root resorption information based on recent *H. sapiens* time ranges (Moorrees et al., 1963).

### 2.4 Morphometric analysis

Orientation of each tooth was performed by using the best‐fit plane computed at the cervical line (i.e., the cervical plane that best fits a spline curve digitized at the cervical line) parallel to the *xy*‐plane of the Cartesian coordinate system (Been et al., 2017; Benazzi et al., 2013; Bocherens & Drucker, 2006; Fiorenza et al., 2018) by using the Geomagic Design X 3D metrology software (3D Systems Software, Rock Hill, South Carolina, USA). The teeth were then rotated around the z‐axis in order to comply with the following criteria: the mesiodistal fissure parallel to the x‐axis and the lingual fissure parallel to the y‐axis. Finally, the mesiodistal (MD) and buccolingual (BL) dimensions (the size of the bounding box enclosing the crown and cervical outlines) of the RSS1 and RSS2 were analyzed. The measurements were then compared to 99 lower deciduous second molars including Neanderthals (N; *n* = 34), early *Homo sapiens* (EHS; *n* = 8) and recent *Homo sapiens* (RHS; *n* = 57), collected from the scientific literature (Hershkovitz et al., 2011) following comparable and previously published protocols (Benazzi et al., 2013; Harvati et al., 2015; Margherita et al., 2016, 2017).

The cervical line of each tooth crown was digitized (Figure S1a) with a ‘spline curve’ in Geomagic Design X software (3D Systems Software, Rock Hill, South Carolina, US) and the best‐fit plane of the cervical line (here, cervical plane) was computed (Figure S1a). The silhouette of the oriented crown outline was then projected onto the cervical plane. The fractured or worn areas of original crown outline were corrected (gray color) by using as reference the buccolingual contour extent. In Rhino v. 4.0 (Robert McNeel & Associates, Seattle, WA), both crown outlines and cervical outlines were centered superimposing the centroids of their area (Figure S1b). All the outlines were represented by 16 pseudo‐landmarks obtained by equiangularly spaced radial vectors out of the centroid. The first radius is directed buccally and parallel to the y axis of the Cartesian coordinate system (Benazzi, Fornai, et al., 2011) (Figure S1c). Finally, size information was oriented and centered with a uniform scaling of the pseudo‐landmark configurations to unit centroid size (Figure S1d).

The chosen shape variables (crown and cervical outlines) were then projected into the shape‐space obtained from a principal component analysis (PCA) of the comparative sample (Table 2) of Neanderthal (*n* = 15), upper Paleolithic *H. sapiens* (*n* = 9) and recent *H. sapiens* (RHS; *n* = 31) used by Margherita et al., 2017 to explore intra‐ and inter‐group relationships.

**TABLE 2:** Comparative sample of lower dm2 used for analyses

Taxon	Specimen	Country	Side	Wear stage (Smith 1984)
N	Abri Suard S14–5	France	R	2
	Abri Suard S37	France	R	1
	Abri Suard S42	France	R	1
	Cavallo A	Italy	L	5
	Couvin	Belgium	R	3
	Engis 2	Belgium	R	3
	Krapina d62	Croatia	L	2
	Krapina d63	Croatia	L	3
	Krapina d64	Croatia	L	3
	Krapina d65	Croatia	L	1
	Krapina d66	Croatia	L	4
	Krapina d68	Croatia	R	1
	Roc de Marsal 1	France	L	1
	Scladina 4A‐13	Belgium	L	6
	Dzu2	Georgia	R	4
UPHS	Dolnı Vestonice 36–6	C.Republic	L	1
	La Madeleine 4	France	L	1
	Paglicci 38	Italy	R	1
	Paglicci 39	Italy	L	3
	Paglicci 40	Italy	R	1
	Paglicci 41	Italy	L	3
	Paglicci 42	Italy	L	6
	Bondi 1	Georgia	R	7
	Lagar Velho 1	Portugal	R	2
RHS	contemporary samplesf	Austria = 14	*L* = 9	wear stage 1 = 21
			*R* = 5	
		France = 7	L	Wear stage 2 = 6
		Italy = 10	L	Wear stage 3 = 4

*Note*: For more information, see Benazzi et al. (2012) and Margherita et al. (2017)

The lateral enamel thickness was computed for the region of the tooth included between the following planes (Figure S1e) (Benazzi, Fornai, et al., 2011; Toussaint et al., 2010): (1) ”cervical plane”; (2) ”cutting plane” (a plane parallel to the cervical plane), which passed through the lowest point of the enamel‐dentine junction (EDJ) in the mid‐occlusal basin (Benazzi, Fornai, et al., 2011), ultimately cutting the occlusal portion of the tooth (called “cutting plane” in Figure S1e). From this portion of the crown, three measurements were collected: the lateral enamel volume (mm^3^; Figure S1f), the lateral dentine plus pulp volume (LDPV in mm^3^; corresponding to the yellow portion in Figure S1f), and the EDJ lateral surface (mm^2^; Figure S1g), which do not consider the fractured side of the dental crown (i.e. distal side of RSS 1) (Benazzi, Fornai, et al., 2011).

These measurements were used for the computation of both the 3D lateral average enamel thickness index (LAET) corresponding to the volume of lateral enamel thickness divided by the EDJ lateral surface (this index is in millimeters) and the 3D lateral relative enamel thickness index (LRET) corresponding to the LAET divided by the cubic root of LDPV (this index is scale‐free) (Martin, 1985; Olejniczak et al., 2008). Finally, LAET and LRET of RSS1 and RSS2 were compared to the comparison sample (*N* = 10; MH = 18) published in Benazzi et al.2012 (Table 3).

**TABLE 3:** List of fossil and extant human dm2s used for lateral enamel thickness

Taxon	Specimen	Country	Wear stage (Smith, 1984)
Neanderthal	Abri Suard S14‐5	France	2
	Abri Suard S37	France	1
	Abri Suard S42	France	1
	Cavallo A	Italy	5
	Couvin	Belgium	3
	Engis 2	Belgium	3
	Krapina d62	Croatia	2
	Krapina d63	Croatia	3
	Krapina d64	Croatia	3
	Roc de Marsal 1	France	1
MH	Lagar Velho 1	Portugal	2
	La Madeleine 4	France	1
	contemporary sample	Austria = 1	Wear stage 1 = 10
		France = 7	Wear stage 2 = 4
		Italy = 8	Wear stage 3 = 2

Abbreviation: MH, Modern Human. For more detail, see Benazzi, Fornai, et al. (2011).

### 2.5 Statistical analysis

We calculated pairwise Euclidean distances on n‐1 PCA coordinates for all individuals and computed a permutational multivariate analysis of variance (PERMANOVA) to assess the presence of significant differences between the three examined groups (N, UPHS and RHS) by considering the variability expressed by all principal components (PCs) at once. We avoided problems related to multiple testing by treating results with Bonferroni correction (Table S2 and S3). To obtain a probabilistic attribution of the two RSS teeth to each of the three reference taxa groups (N, UPHS, RHS) we performed and compared the performance of three supervised learning algorithms on both crown and cervical outlines, since – when taken individually – they may lead to different taxonomic attributions (Harvati et al., 2015; Zanchetta et al., 2018). More specifically, we selected the first eight principal components (PCs) accounting for 92% and 95% of crown and cervix data total variance, respectively (Bailey et al., 2020; Jolliffe, 2002; Oxilia et al., 2018; Sorrentino et al., 2020). We tested for normality (Shapiro–Wilk test) and homogeneity of variances across groups (Fligner‐Killeen tests) on each PC. Since we found violations of both assumptions in crown data (Tables S4 and S5) and heteroscedasticity in cervix data (Tables S6 and S7), we used flexible discriminant analysis (FDA), a flexible extension of linear discriminant analysis (LDA) that uses nonlinear combinations of predictors allowing for a low misclassification error when modeling non‐linear, non‐normal, and non‐homogeneous data (Hastie et al., 1994; Hastie, Hastie et al., 2009) (see Supplementary Information). We also used a multiadaptive regression splines (MARS) model (Friedman, 1991; Ruppert, 2004). This algorithm identifies the value intervals that the best discriminate between groups by iteratively running linear regressions for each group and finding the predictor points that minimize within‐group total error (*knots*). These points are then used to link individual linear functions into the final model (Friedman, 1991; Hastie et al., 2009). We controlled for overfitting of the models using generalized cross‐validation (GCV), a stepwise process which assesses the ratio between the goodness of fit of the model and the number of parameters (*knots*; Supplementary Information). We then tested the performance of FDA and MARS against a Random Forest (RF) classifier (Liaw & Wiener, 2002). The latter uses recursive binary splitting to grow classification trees carrying out a multiple sampling with replacement at each node and choosing the most commonly occurring model among all predictions based on the sampled subsets (Supplementary Information). We validated each model (FDA, MARS, and RF) with a repeated 10‐fold cross‐validation (Supplementary Information). The data were processed and analyzed using R v.4.1.0 (R Core Team, 2021), and all details on functions and packages, including data templates and code for replicating all the analyses presented here are available at http://doi.org/10.6092/unibo/amsacta/6724.

## 3 RESULTS

### 3.1 Chronology

New radiocarbon dates of the Uluzzian (F14 t19) and Late Mousterian (F14 t39) layers at Roccia San Sebastiano were obtained for the relevant layers. These dates are not directly comparable with the already available chronology for the archeological sequence (Aiello et al., 2018) since the latter was not obtained through ultrafiltration protocol (Brock et al., 2007). The new chronological range obtained for the Uluzzian layer (trench F14 spit t19; ETH‐99090.1.1) is 42,640–42,380 cal BP, while the Mousterian layer (trench F14 spit t39; ETH‐99085.1.1) localized at the bottom of the stratigraphic sequence below the layer in which RSS1 was found, dates to 44,810–44,230 cal BP. Both results are reported at 68.2% probability (Table 1) and show no overlap between the two phases of occupation. However, we will be investigating the chronology further with additional samples.

### 3.2 Morphologic and morphometric analysis

#### 3.2.1 RSS1

**FIGURE 2:**
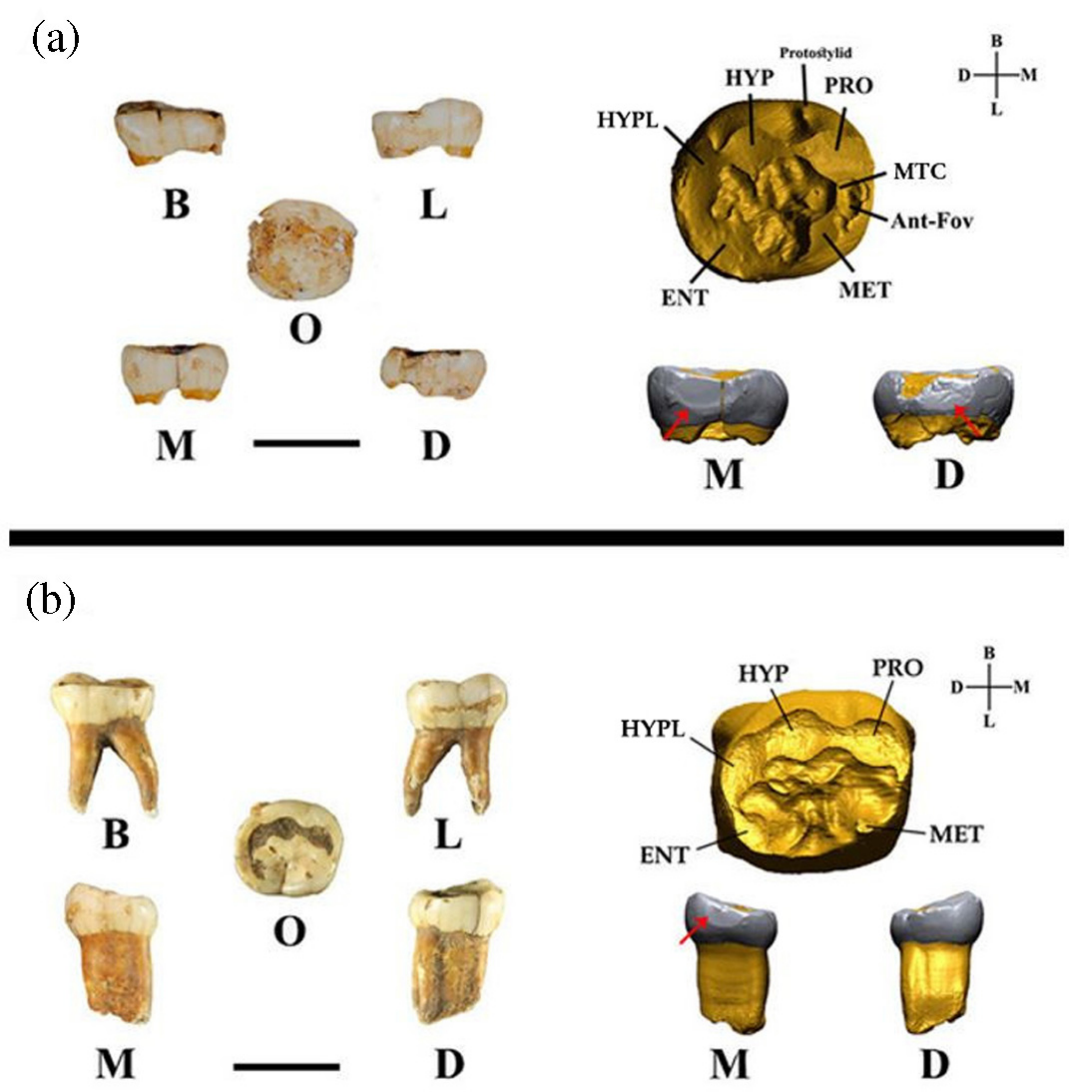
Three‐dimensional digital models of lower left second deciduous molar, Ldm2; A) RSS 1 and B) RSS 2. On the right side, the enamel‐dentine junction (EDJ) of the teeth virtually analyzed. Ant‐Fov, anterior fovea; MTC, middle trigonid crest; MET, Metaconid; ENT, Entoconid; HYP, Hypoconid; HYPL, Hypoconulid; PRO, Protoconid; B, buccal; D, distal; L, lingual; M, mesial; O, occlusal. The horizontal black bars are equivalent to 1 cm

RSS1 is a worn (wear stage 5) (Molnar, 1971) lower left second deciduous molar (Ldm2) with a complete crown and less than a quarter of the root preserved (Figure 2a). More specifically, root resorption is at a Res3/4 stage (Moorrees et al., 1963) suggesting that the tooth had been lost antemortem, at an age ranging from 9 to 12 years old.

From the occlusal view, the crown outline shows a bucco‐distal enlargement and a convex lingual side. Most dental features on the external enamel surface were removed by wear, but on the enamel‐dentine junction (EDJ), five principal cusps and a weak anterior fovea bordered distally by a weak (stage = 3) (Bailey et al., 2011) mid‐trigonid crest (MTC) were observed. The latter are typically observed in Neanderthal but also a low frequency in *H. sapiens* molars deciduous (Bailey, 2017). On the buccal side a protostylid with a positive free apex is evident and developed close to the buccal groove (Turner et al., 1991). Interproximal facets are clear both mesially (length = 4.29 mm; height = 2.26 mm) and distally (length = 3.69 mm; height = 2.17 mm), but their size is underestimated due to occlusal wear and, for the distal side, the formation of a post‐depositional fracture. The tooth crown has an MD diameter of 9.63 mm (minimum estimation due to the interproximal wear) and a BL diameter of 9.03 mm. At the cervix the MD diameter is 8.25 mm and the BL diameter is 7.58 mm (see Supplementary Information).

#### 3.2.2 RSS2

RSS2 is a worn (wear stage 5) (Molnar, 1971) Ldm2 with a complete crown, the entire root preserved and an open apical foramen (Figure 2b). Specifically, the root suggests that the tooth was lost post mortem at an age ranging from 4 (tooth in occlusion) to 6 years (absence of distal interproximal wear facet) (Moorrees et al., 1963).

From the occlusal view, the crown outline shows a bucco‐distal narrowing and a concave lingual side. Most of the crown's occlusal morphology was removed by wear, but on the EDJ, five principal cusps are present. The occlusal topography of the EDJ is characterized by several crests reaching the central basin from the cusps tips (protoconid, hypoconulid) and lingual margin.

An interproximal facet is clearly visible mesially (length = 3.61 mm; height = 1.76 mm). On the distal side of the occlusal surface several chips were observed, in particular on the entoconid, hypoconid and hypoconulid. On the mesial side, we identified only one chip closer to the interproximal wear facet. The tooth crown has a MD diameter of 10.12 mm (minimum estimation due to wear) and a BL diameter of 9.16 mm. At the cervix the MD diameter is 7.89 mm and the BL diameter is 7.08 mm (see Supplementary Information).

### 3.3 Taxonomic attribution

#### 3.3.1 RSS1

No difference exists in the distributions of either the crown or cervical outlines between UPHS and RHS (Tables S2 and S3). We therefore grouped all *Homo sapiens* specimens in our reference sample into a single class (MH) and measure the probability of RSS1 and RSS2 of being attributed to either MH or Neanderthals (N). After repeated 10‐fold cross‐validation, FDA is the best performing algorithm for both crown and cervix data (Accuracy_crown_ = 0.94; Accuracy_cervix_ = 0.95), followed by MARS (Accuracy_crown_ = 0.93; Accuracy_cervix_ = 0.93) and RF (Accuracy_crown_ = 0.92; Accuracy_cervix_ = 0.9).

**FIGURE 3:**
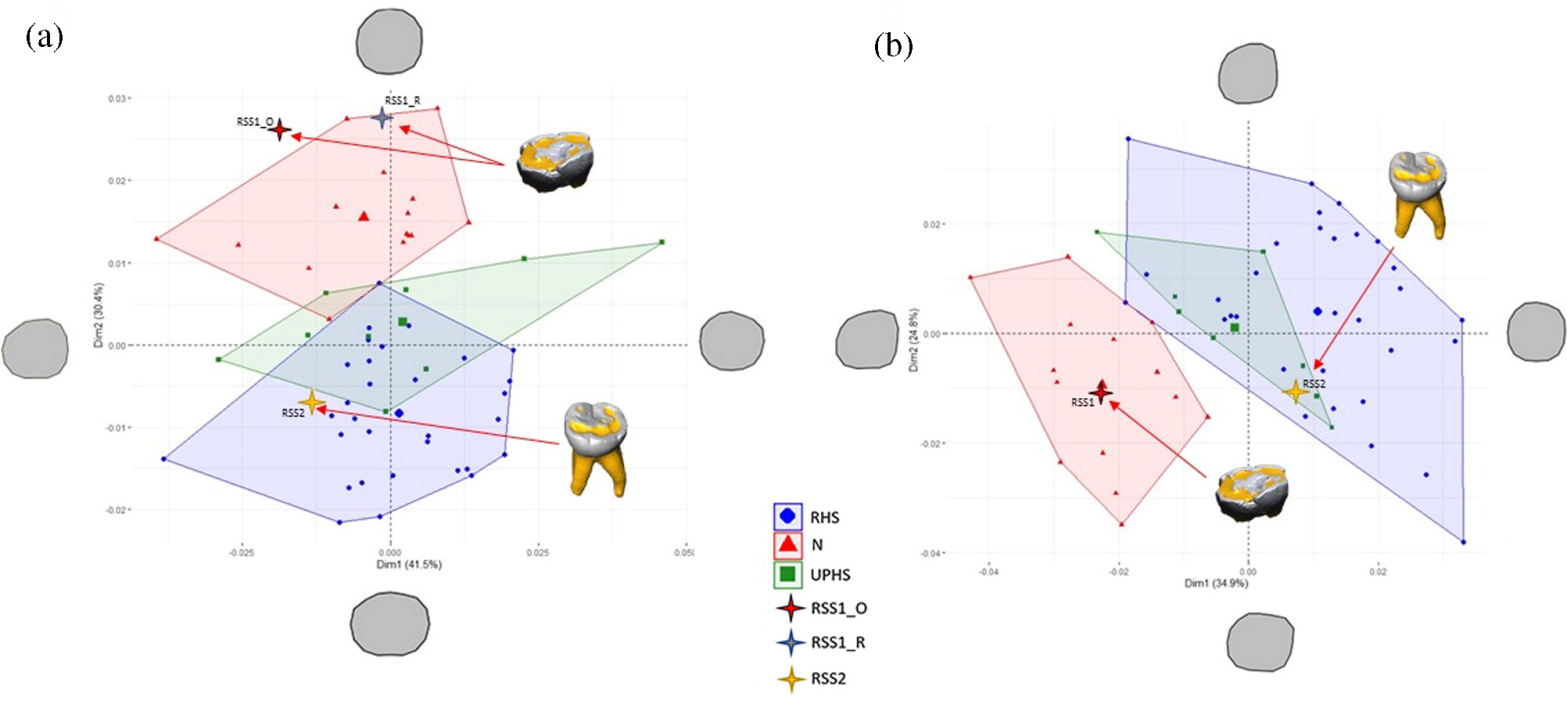
Shape–space principal component analysis (PCA) plots of Ldm2 crown (a) and cervical (b) outlines. The deformed mean crown outline in the direction of the PC is drawn at the extremity of each axis. RSS1_O, Roccia san Sebastiano 1 original; RSS1_R, Roccia san Sebastiano 1 restored; N, Neanderthal; RHS, recent Homo sapiens; UPHS, upper Paleolithic Homo sapiens

**FIGURE 4:**
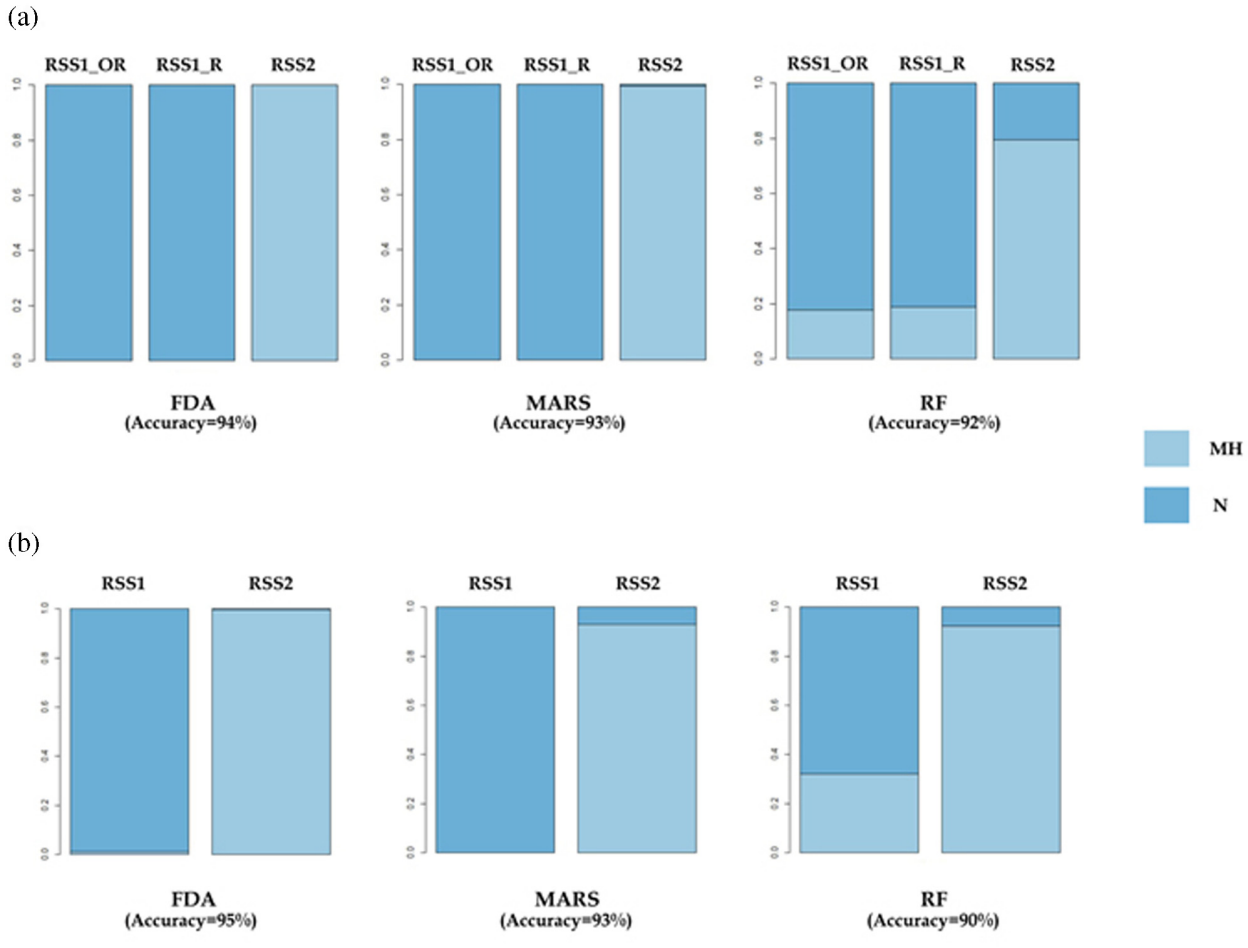
Taxonomic attribution obtained from supervised learning models on RSS1 and RSS2 crown (a) and cervical (b) outlines data. RSS1_OR, RSS1 original; RSS1_R, RSS1 restored; FDA, flexible discriminant analysis; MARS, multivariate adaptive regression splines; RF, random forest; MH, modern humans; N, Neanderthals

As far as RSS1 is concerned, crown and cervical outlines of both the original and the restored tooth fall within the Neanderthal range of variability (Figure 3). All classification algorithms coherently assign both original and restored RSS1 crown outline to Neanderthals, and the same result is obtained for the original cervical outline (Figure 4; Table S8). Taxonomic attribution is also supported by lateral enamel thickness analysis, where RSS1 lateral average and relative enamel thickness (LAET and LRET, respectively) are closer to Neanderthal values than to the *H. sapiens* mean (Table S9).

No difference exists in the distributions of either the crown or cervical outlines between EHS and RHS (Tables S2 and S3). Both crown and cervical outlines of RSS1 fall within the Neanderthal range of variability (Figure 3).

#### 3.3.2 RSS2

Turning to RSS2, both crown and cervical outlines fall within the *H. sapiens* range of variability (Figure 3), and are consistently attributed to MH by all classification algorithms (Figure 4, TableS8). Lateral Relative Enamel Thickness (LRET) for RSS2 is considerably higher than mean values for both MH and N (Table S10), making it difficult to interpret it in terms of taxonomic attribution.

## 4 DISCUSSION

### 4.1 Biological inferences

In this study, we show the most recent direct evidence of human remains (RSS1) belonging to a Neanderthal, who lived during the middle‐to‐upper Paleolithic transition occurrig across Italy (44,810–44,230 cal BP). Morphological analysis of RSS1 documents a bucco‐distal enlargement and a convex lingual side (from the occlusal view), as well as a complex morphology of the EDJ in the occlusal aspect (i.e. protostylid, anterior fovea and mid‐trigonid crest). As pointed out above (see also Figure 2a), these features are typically observed in Neanderthals (Williams et al., 2018) and allow RSS1 to be assigned to this human group. This result is confirmed by morphometric analysis of coronal diameters (Table S12) and outlines (crown and cervical) that corroborate the taxonomic attribution, as well as by the posterior probabilities obtained by all the supervised classification algorithms used in this work (Figure 4, Table S8). Moreover, the layer in which RSS1 was found appears more recent than spit t39 that we dated to 44,810–44,230 cal BP (68.3% probability). These results allowed us to confirm the most recent evidence in Southern Italy.

In this study we also provide the oldest date for the Uluzzian techno‐complex in the Tyrrhenian side of the Italian Peninsula (42,640–42,380 cal BP) documenting another evidence of Uluzzian occupation. Similarly to Pleistocene (Williams et al., 2018) and recent human samples (Bailey et al., 2011; Margherita et al., 2017; Williams et al., 2018), RSS2 has a crown (Table S13) outline characterized by bucco‐distal narrowing, straighter lingual side, and a complex morphology in the occlusal aspect of the EDJ (i.e. crests in the mid‐occlusal basin, but absence of MTC) (Figure 2b). Indeed, as confirmed by all state of the art classification algorithms used, we can state that this tooth belongs to *Homo sapiens* (Figure 4, Table S8).

### 4.2 Techno‐cultural observations

Understanding the dynamics of contact between species is crucial, especially investigating at the middle‐to‐upper Paleolithic transition. Several questions are indeed still unsolved regarding the mode and tempo of Neanderthals extinction and the following success of *Homo sapiens*. As far as Italy is concerned, there are still open questions regarding the relationship between Mousterian and Uluzzian techno‐complexes across the peninsula, and how these may or may not relate to different human species, in particular considering the homogeneity of the Uluzzian techno‐complex throughout Italy despite environmental and chronological differences documented between different areas.

Mousterian and Uluzzian techno‐complexes in Italy have been documented in some cave and sheltered sites (for an updated review), such as Broion (Peresani et al., 2019), Fumane (Peresani et al., 2016), La Fabbrica (Villa et al., 2018), Castelcivita (Arrighi, Marciani, et al., 2020; Gambassini, 1997), Cala (Benini et al., 1997); Uluzzo C (Silvestrini et al., 2021), and Cavallo (Fabbri et al., 2016; Moroni et al., 2013). However, only a few human remains dated between 50 and 40 ka have been discovered in Italy (Buzi et al., 2021) and it is notable that the association between one fossil in one context does not imply that one techno‐complex is exclusively related with one or the other species. To date, all the Neanderthal human remains are constrained between 50 and 45 ka cal BP, as the most recent being an incisor from Cavallo cave dated to 45 ka ago (Moroni et al., 2018). At Cavallo the stratigraphic sequence suggests *Homo sapiens* were already present in southern Europe at least since 45–43 ka cal BP (Cavallo B ‐ left dP3, and Cavallo C ‐ left dP4) (Benazzi, Douka, et al., 2011) and spread to other parts of Italy at least by ~41–40 ka cal BP as documented in Fumane cave (Fumane 2 – right di^2^) and Bombrini (Bombrini tooth ‐ left di_2_) (Benazzi et al., 2015).

At RSS we show that human remain RSS1 belong to a Neanderthal, and RSS2 belongs to *Homo sapiens*. RSS1 (the Neanderthal specimen) was discovered in association with Mousterian materials. The layer where RSS2 (*Homo sapiens*) was discovered contains all the main features of the Uluzzian lithic assemblage (Collina, Benazzi, et al., 2020; Collina, Marciani, et al., 2020) (see archeological setting). However, the current data are provided only based on the trenches; thus, the only future excavated extensions will shed light on the dynamics of occupation of the site and possible post‐depositional process as well as provide additional radiocarbon dating of the stratigraphic sequence to further specify this chronological attribution. As a matter of fact, in the Uluzzian layer in trench E16, there are some Gravettian tools possibly filtered from the overlying layer. However, the lithic assemblage of this particular layer consistently exhibits the main features of Uluzzian technology, in spite of the presence of a very limited Gravettian infiltration (22 items on a totality of 3257 items) (Collina et al. 2020). The attribution of this Uluzzian deposit to the period 42,640–42,380 cal BP makes it the earliest documented evidence of this techno‐complex on the western side of the Italian Peninsula.

More in detail, we note that at Roccia San Sebastiano the replacement of the Mousterian by the Uluzzian occurred over the same range of time (~ 2000 years) as at Grotta del Cavallo (Apulia, southeaster Italy) (Benazzi, Fornai, et al., 2011; Sarti & Martini, 2020). However, this cultural replacement at RSS occurred almost 2000 years later than the Apulian site, in agreement with the fact that the oldest Uluzzian evidence is known so far in layer EIII of Grotta del Cavallo (about 44 ka BP).

## 5 CONCLUSION

Our study provides the evidence of the latest fossil evidence for a Neanderthal presence in Italy (44,800–44,230 cal BP) and the earliest presence of Uluzzian groups in the Tyrrhenian side of the Italian Peninsula. The chronology of the Uluzzian assemblage (42,640–42,380 cal BP) of Roccia San Sebastiano is older than the one measured for the uppermost Uluzzian layer at Castelcivita (41,910–40,570 cal BP) (Douka et al., 2014; Wood et al., 2012). This supports a later expansion of Uluzzian groups from the core area in Apulia (Marciani et al., 2020), where the oldest Uluzzian settlement was found at Grotta del Cavallo, (ca. 45–43 ka cal BP) (Benazzi, Douka, et al., 2011; Moroni et al., 2018) to the area of Roccia San Sebastiano (Figure S2). Present results confirm that Southern Italy, and Roccia San Sebastiano in particular, is a key region to disentangle the biocultural dynamics of the two human groups.

## AUTHOR CONTRIBUTIONS

**Gregorio Oxilia:** Conceptualization (lead); formal analysis (lead); investigation (lead); methodology (lead); software (equal); supervision (equal); validation (equal); visualization (lead); writing – original draft (lead); writing – review and editing (equal). **Eugenio Bortolini:** Methodology (equal); supervision (equal); validation (equal); writing – original draft (equal); writing – review and editing (equal). **Giulia Marciani:** Formal analysis (equal); investigation (equal); supervision (equal); validation (equal); visualization (equal); writing – original draft (equal); writing – review and editing (equal). **Jessica Cristina Menghi Sartorio:** Formal analysis (equal); methodology (equal); supervision (equal); writing – original draft (equal); writing – review and editing (equal). **Antonino Vazzana:** Methodology (equal); visualization (equal); writing – review and editing (equal). **Matteo Bettuzzi:** Methodology (equal); writing – review and editing (equal). **Daniele Panetta:** Methodology (equal); visualization (equal); writing – review and editing (equal). **Simona Arrighi:** Writing – review and editing (equal). **Federica Badino:** Supervision (equal); writing – review and editing (equal). **Carla Figus:** Writing – review and editing (equal). **Federico Lugli:** Writing – review and editing (equal). **Matteo Romandini:** Writing – review and editing (equal). **Sara Silvestrini:** Writing – review and editing (equal). **Rita Sorrentino:** Writing – review and editing (equal). **Adriana Moroni:** Supervision (equal); validation (equal); writing – review and editing (equal). **Carlo donadio:** Formal analysis (equal); software (equal). **Maria Pia Morigi:** Methodology (equal); writing – review and editing (equal). **viviane Slon:** Formal analysis (equal); investigation (equal); methodology (equal). **Marcello Piperno:** Data curation (lead); resources (lead); writing – original draft (equal); writing – review and editing (equal). **Sahra Talamo:** Investigation (equal); methodology (equal); supervision (equal); writing – original draft (equal); writing – review and editing (equal). **Carmine Collina:** Data curation (lead); investigation (equal); resources (lead); writing – original draft (equal); writing – review and editing (equal). **Stefano Benazzi:** Conceptualization (lead); data curation (lead); investigation (lead); supervision (lead); validation (lead); writing – original draft (lead); writing – review and editing (lead).

## CONFLICT OF INTEREST

The authors declared that there is no conflict of interests.

## Supporting information


**Figure S1** Sequence of lateral enamel thickness analysis.
**Figure S2.** Paleogeographic map of Italy. Presence of Uluzzian groups at around 43–45 ka cal BP and on the Tyrrenian coast at around 40 ± 1.6 ka Optically stimulated luminescence (OSL).
**Figure S3.** RSS2 (lower left second deciduous molar). Six views comprehensive of the root after sampling in the centre (P). O, Occlusal; P, periapical; B, buccal; D, distal; L, lingual; M, mesial. Scale bar: 2 mm
**Figure S4.** RSS2 (lower left second deciduous molar). Five comprehensive views of the tooth after restoration. P, periapical; B, buccal; D, distal; L, lingual; M, mesial. Scale bar: 2 mm
**Table S1.** Stratigraphic sequence correlation.
**Table S2.** Permutational multivariate analysis of variance test (with Bonferroni correction) of the crown outlines between recent *Homo sapiens* (RHS), upper Paleolithic *Homo sapiens* (UPHS) and Neanderthals (N).
**Table S3.** Permutational multivariate analysis of variance test (with Bonferroni correction) of the cervical outlines between Recent *Homo sapiens* (RHS), upper Paleolithic *Homo sapiens* (UPHS) and Neanderthals (N).
**Table S4.** Shapiro–Wilcoxon normality test for crown outlines
**Table S5.** Fligner‐Killeen homogeneity of variance test for crown outlines
**Table S6.** Shapiro–Wilcoxon normality test for cervical outlines
**Table S7.** Fligner‐Killeen homogeneity of variance test for cervical outlines
**Table S8.** Posterior Probabilities obtained from supervised learning models on crown and cervix data.
**Table S9.** Roccia San Sebastiano 1 (RSS1) lateral enamel thickness measurements of individuals belonging to the comparative sample.
**Table S10.** Roccia San Sebastiano 2 (RSS2) lateral enamel thickness measurements of individuals belonging to the comparative sample.
**Table S11.** Results of the DNA analysis of RSS1.
**Table S12**. Roccia San Sebastiano 1 (RSS1) buccolingual crown diameters (in mm) of the comparative sample.
**Table S13.** Roccia San Sebastiano 2 (RSS2) buccolingual crown diameters (in mm) of the comparative sample.

## Data Availability

Data openly available in a public repository that issues datasets with DOIs 10.6092/unibo/amsacta/6629, reference number 6629. The sequencing data generated was deposited in the European Nucleotide Archive (accession number PRJEB43838).
