# Supplementary material for: Direct evidence that late Neanderthal occupation precedes a technological shift in southwestern Italy
Source: Am J Biol Anthropol. 2022 Jul 20;179(1):18–30. doi: 10.1002/ajpa.24593 (PMC9541503; doi:10.1002/ajpa.24593)
Supplement: Supplementary file 1 — Figure S1 Sequence of lateral enamel thickness analysis. Figure S2. Paleogeographic map of Italy. Presence of Uluzzian groups at around 43–45 ka cal BP and on the Tyrrenian coast at around 40 ± 1.6 ka Optically stimulated luminescence (OSL). Figure S3. RSS2 (lower left second deciduous molar). Six views comprehensive of the root after sampling in the centre (P). O, Occlusal; P, periapical; B, buccal; D, distal; L, lingual; M, mesial. Scale bar: 2 mm Figure S4. RSS2 (lower left second deciduous molar). Five comprehensive views of the tooth after restoration. P, periapical; B, buccal; D, distal; L, lingual; M, mesial. Scale bar: 2 mm Table S1. Stratigraphic sequence correlation. Table S2. Permutational multivariate analysis of variance test (with Bonferroni correction) of the crown outlines between recent Homo sapiens (RHS), upper Paleolithic Homo sapiens (UPHS) and Neanderthals (N). Table S3. Permutational multivariate analysis of variance test (with Bonferroni correction) of the cervical outlines between Recent Homo sapiens (RHS), upper Paleolithic Homo sapiens (UPHS) and Neanderthals (N). Table S4. Shapiro–Wilcoxon normality test for crown outlines Table S5. Fligner‐Killeen homogeneity of variance test for crown outlines Table S6. Shapiro–Wilcoxon normality test for cervical outlines Table S7. Fligner‐Killeen homogeneity of variance test for cervical outlines Table S8. Posterior Probabilities obtained from supervised learning models on crown and cervix data. Table S9. Roccia San Sebastiano 1 (RSS1) lateral enamel thickness measurements of individuals belonging to the comparative sample. Table S10. Roccia San Sebastiano 2 (RSS2) lateral enamel thickness measurements of individuals belonging to the comparative sample. Table S11. Results of the DNA analysis of RSS1. Table S12. Roccia San Sebastiano 1 (RSS1) buccolingual crown diameters (in mm) of the comparative sample. Table S13. Roccia San Sebastiano 2 (RSS2) buccolingual crown diameters (in mm) of the comparative s [file AJPA-179-18-s001.docx]

**Supplementary Information**

Direct evidence that late Neanderthal occupation precedes a technological shift in southwestern Italy

***Environmental Setting***

During this period, sub-millennial environmental changes can be inferred from Mediterranean palaeoecological records (Allen et al., 1999; Allen et al. 2000; Fletcher et al., 2010), which show recurrent successions of woodland and more open conditions, broadly recalling Greenland Stadial /Interstadial cyclicity. In this framework, Greenland Stadial related to Heinrich Stadials 5 (49 – 47 ka) and 4 (40.2-38.3 ka) (Sanchez Goñi & Harrison, 2010) were characterized by enhanced open and dry conditions on land (Margari et al. 2009; Tzedakis, 1999; Tzedakis et al. 2006), although their impact seems to have been negligible in the Apulia region based on Pozzo Cucu’ Cave isotopic records (Columbu et al., 2020). Also, the Campanian Ignimbrite eruption (^40^Ar/^39^Ar age: 39.85 ± 0.14 ka) (Giaccio et al. 2017), whose spread deposit outcrop in the area of Mondragone (Aiello et al., 2018; Kono, 2004; Pennetta et al., 2016) had, presumably, further impacts in southern Italy and especially in proximal sites such as Roccia San Sebastiano. This could be supposed since the site is located only ⁓50 km north of the Campi Flegrei (Lowe et al., 2012) active volcanic area.

***The site: stratigraphic sequence***

The site of Roccia San Sebastiano was discovered in December 1999 during systematic surveys carried out by the Second University of Naples within a project promoted by the Prehistory Chair of the University of Naples ‘Federico II’. Since 2001, systematic excavations were conducted by the Museo Civico Biagio Greco of Mondragone under the direction of Marcello Piperno and Carmine Collina (Belluomini et al. 2007; Collina et al. 2008; Collina et al. 2007; Collina et al. 2011, 2018; Collina et al., 2020).

Excavations carried out from 2001 to 2010 (led by M. Piperno) were mainly focused on removing the sediment sealing the entrance of the cave and the reworked sediments inside the cave. In 2003 the first *in situ* level containing a Gravettian occupation (sub-unit C) (Collina et al. 2008; Collina et al. 2007) was discovered in an area of 6 m^2^ (squares F-E 10, 11, 12). This is the only level that has been excavated in the extension.

The archaeo-stratigraphic description, which is the reference for the archaeological deposit of the cave, is defined first in a test trench 2x1m located in squares in E14–E15. This trench reaches 2.8 m in depth and ends with a sterile layer. Two further trenches were dug (excavation led by C. Collina), one in square F14, 1x1x2.8m in depth, and the other in square E16, 1x1x2.15m in depth which is still under excavation (Table S1). The teeth come from spit t34 in E14–E15 trench (Late Mousterian; RSS1) and spit t16 in E16 trench (Uluzzian; RSS2). No tephra layers have been discovered inside the cavity.

The archaeological sequence dug in trench E14–15 at Roccia San Sebastiano can be divided into three main units (labelled Unit 1 to 3) which were based on their overall stratigraphic features and on the archaeological materials that were discovered within. Unit 1 (sub-units: C-Ca -recent Gravettian, Cb-Gravettian with Noailles burins, and Cc-Early Gravettian) consists of brownish sandy silt deposits with occasional carbonate concretions. The uppermost portion of the unit displays a coarser matrix, while at the base, traces of charcoal levels and carbonate cobbles occur (Collina et al., 2020). Unit 2 (sub-units: Cd-Initial Gravettian, and Ce-Aurignacian Dufour) consists of reddish-brown sandy silt deposits with the occasional presence of limestone pebbles. The sediments slope slightly towards the interior part of the cave (Collina et al., 2020). Unit 3 (sub-units: Cf-Uluzzian and Cg- Mousterian) mainly consists of dark sandy, silty deposits, rich in organic matter and with abundant remains of fauna. In greater detail, the upper portion of unit 3-sub-unit Cg is made up of two lithostratigraphic strata; the upper is made of reddish compact clay with scarce limestone debris, whilst the lower is mainly composed of yellowish clay (Collina et al., 2020) (Table S1, Figure 1). To note, this description is based on the trench E14-15 (Collina et al. 2011). The Unit 2 (sub-units: Cd and Ce respectively Initial Gravettian and Aurignacian Dufour) is abset in the two new trenches (F14, E16).

***The site: lithic assemblages***

The Gravettian assemblage (Unit 1-sub-units: C-Ca, Cb, Cc; Unit 2-Cd) is characterised by the use of mainly local raw materials primarily composed of chert, followed by radiolarite, quartzite and sandstone. Pebbles of small and medium size were collected from the fluvial deposit near the site. However, it is attested that a certain number of tools were manufactured in raw materials (Collina et al. 2008). The production is mainly characterised by a unidirectional volumetric debitage aimed at producing bladelets and blades. The assemblage displays a highly specialized character, as it is especially aimed at producing micro-gravettes and a large quantity of burins. End-scrapers, side scrapers and splintered pieces are also present (Collina et al. 2008).

The assemblage defined as Aurignacian Dufour (Unit 2-Ce) is an industry characterised by Aurignacian features, rich in typical Dufour bladelets and pieces with marginal retouch. The production of bladelets with a trapezoidal cross-section is made from small cores made on pebbles. The presence of several bone artefacts is also attested (Collina et al. 2011).

The lithic assemblage of the Unit 3-Cf was recently attributed to the Uluzzian because it shows all the main technical feature of the Uluzzian techno-complex (Marciani et al., 2020; Moroni et al., 2018; Riel-Salvatore, 2009). Namely, it exploits principally block of raw material, which naturally already present the convexities, angles and guide ribs appropriate for knapping; thus, it implies rough management of the striking platforms and convexities, indeed striking platform are cortical or opened by a single or few removals, and the debitage surfaces are simply managed. The reduction sequences follow unidirectional, bidirectional, or orthogonal directions. The aims of the debitage are small blades and small flakes showing a low degree of standardisation due to the extensive use of the bipolar technique on anvil. There is an absence of integrated concepts of debitage such as levallois or discoid. A presence of lunates and end-scrapers among the retouched tools is noted (Collina et al., 2020). Moreover, the study of the material of Unit 3-Cf of Roccia San Sebastiano helped to refine some conceptual point on this challenging techno-complex, namely: i) the conceptualisation of Uluzzian production seems “simple”, but actually, it is a straightforward method to obtain the target objects. (ii) The bipolar percussion on anvil is a deliberate choice used in the same reduction sequence of direct percussion. (iii) This type of production allows the obtaining of several items with sharp edges and rectilinear profile, which could be useful in realising composite tools (Collina et al., 2020) possibly projectiles (Moroni et al., 2018; Riel-Salvatore, 2009; Sano et al., 2019).

The rich Mousterian assemblage (Unit 3-Cg) is characterised by a dominance of Levallois debitage with blanks showing facetted and dihedral butts. Retouched tools are mainly composed of points, side scrapers and notches (Collina et al. 2011).

***Radiocarbon dating***

The samples are decalcified in 0.5M HCl at room temperature until no CO_2_ effervescence is observed. 0.1M NaOH is added for 30 minutes to remove humics. The NaOH step is followed by a final 0.5M HCl step for 15 minutes. The resulting solid is gelatinised following Longin, 1971 at pH 3 in a heater block at 75°C for 20h. The gelatine is then filtered in an Eeze-Filter™ (Elkay Laboratory Products Ltd, UK) to remove small (>80 μm) particles. The gelatine is then ultrafiltered^11^ with Sartorius ‘VivaspinTurbo’ 30 KDa ultrafilters. Prior to use, the filter is cleaned to remove carbon containing humectants (Brock et al. 2007). The samples are lyophilised for 48 hours. C:N atomic ratios and collagen yields were measured to determine the extent of collagen preservation. Samples were graphitised and dated by accelerator mass spectrometry (AMS) at the ETH Zurich (ETH code). The resulting dates were corrected for a residual preparation background estimated from pretreated ^14^C-free bone samples, kindly provided by D. Döppes (MAMS, Germany) (Korlević et al. 2018). We used the MIni CArbon DAting System (MICADAS, Switzerland) (Wacker et al. 2010) at ETH-Zurich to produce the graphite and to date the samples.

***DNA analysis***

Dentine powder was collected from RSS1 (Table S11) following the removal of a thin layer of surface material. A sub-sample of ca. 5 mg powder was then transferred to a fresh tube, and 500 µl of lysis buffer were added both to the powder in the fresh tube and to the leftover powder (<1 mg) in the original tube. DNA was extracted from one 150–µl aliquot of each lysate with the automated procedure described in Rohland et al. (2018) using binding buffer ‘D’. The extracts were converted into single-stranded, double-indexed DNA libraries (Gansauge et al. 2020) . The number of molecules in the DNA libraries and the yields of an artificial oligonucleotide spiked in them for quality control were assessed by quantitative PCR (qPCR) (Gansauge et al. 2020, Glocke & Meyer et al. 2017). One aliquot of each library underwent targeted enrichment for human mitochondrial DNA (mtDNA) fragments (Maricic et al. 2010, Slon et al. 2017). Shotgun sequencing and sequencing after targeted enrichment were performed on a MiSeq platform (Illumina) in 76-cycles paired-end runs. Forward and reverse reads were collapsed using leeHom (Renaud et al. 2017) and BWA (Li & Durbin 2009) with ‘ancient’ parameters (Meyer et al. 2012) being used to map the sequenced fragments to the reference human genome (hg19 for shotgun data and the revised Cambridge Reference Sequence for enriched mtDNA data). PCR duplicates were removed using bam-rmdup (<https://github.com/mpieva/biohazard-tools/>).

Two DNA libraries were generated from dentine powder collected from the RSS1 tooth. The DNA library prepared from powder that was left-over in the original collection tube contained more molecules than the associated negative controls, indicating a successful library preparation; while the number of molecules in the library prepared from the powder that had been transferred to a fresh tube falls within the range of controls. The numbers of oligonucleotides spiked into both libraries show no deviation from the controls, suggesting that the conversion of DNA fragments into library molecules was not hindered by inhibitory substances (Table S11).

Following shotgun sequencing, 0.5% and 23.6% of fragments longer than 35 base-pairs (bp), respectively, mapped to the human reference genome with a mapping quality of 25 or more. To assess whether the DNA retrieved was of ancient origin, we evaluated the frequency at which fragments starting or ending at a position where the reference carries a cytosine (C) displays a thymine (T). These terminal C to T substitutions are thought to derive from the deamination of cytosines over time and are considered to be indicative of ancient DNA (Briggs et al. 2007). None of these fragments from the first library displayed C to T substitutions. In the second library, only 0.8% and 0.4% did so on their 5’- and 3’-ends, respectively (Table S11). Low frequencies of terminal C to T substitutions were also observed for the mtDNA fragments retrieved following targeted enrichment (5.7% and 3.9% in the first library and 1.5% and 0.9% in the second; (Table S11). These indicate that we were unable to detect traces of endogenous DNA (nuclear or mitochondrial) in RSS1.

***Morphometric analysis (BL and ML Diameters)***

Buccolingual (BL) diameter of RSS1 shows values closer to the Neanderthal mean then to Upper Palaeolithic *Homo sapiens* and same as above expected values (Table S12). At the same time, the BL diameter of RSS2 is equally close to both Neanderthal and Upper Palaeolithic *H. sapiens* expected values*,* but out of the range measured for recent *H. sapiens* (Table S13).

**Tooth sampling and restoration** **of RSS2**

The restoration of RSS2 has been performed by following seven stages as follows: μCT images (see Methods section in the main text) of the original tooth were acquired at the Department of Physics and Astronomy of the University of Bologna and then virtually segmented using Avizo v. 9.2 software (Thermo Fisher Scientific, Waltham) as well as dental tissues (enamel, dentine and pulp chamber) converted to mesh using Geomagic Design X (3D Systems Software, Rock Hill).

Once scanned, the tooth was mechanically split (Figure S3) at the Clean lab of the Department of Cultural Heritage of Ravenna in an environment of ultra-low levels of background contamination, in order to obtain and analyse two root fragments (mesial and distal). From the invasive sampling, 260 mg of powder was gathered for radiocarbon dating and DNA analysis.

The post-sampled tooth was then acquired by microCT at the Department of Physics and Astronomy of the University of Bologna and then virtually segmented using Avizo v. 9.2. The 3D surfaces of the tooth were then created in Geomagic Design X. The 3D surfaces were further processed in the mentioned software for correction of defects (e.g., filling of small holes), to create fully closed surfaces necessary for further analysis (Vazzana et al. 2018, Nowaczewska et al. 2021).

After obtaining the digital models of the tooth before and after sampling, three different spline curves were digitized at the artificial cutting line (one on the external cut line and two long root canals of the after-sampling tooth) to isolate the cutting surface (Benazzi et al. 2014a). The same curves were projected on the digital model of the whole tooth (pre-sampling) to isolate the sampled part from the preserved one. Then a single mesh was created. Finally, a Boolean subtraction was performed between the two meshes (root to be integrated and preserved part of the tooth) to optimize the contact surface. An exact replica of the sampled part was produced again by rapid prototyping technology (LCD Stereolithography) using Orange 10 LCD 3D printer. The prototype was produced using Longer UV resin, layer thickness 0.02 mm, UV Matrix 405nm LED lighting sources and slicing software Longerware. Finally, the root was painted and pasted by using compatible and reversible glues (Figure S4).

**Flexible discriminant analysis and multiadaptive regression splines**

Recent research has established that linear discriminant analysis (LDA) can be performed as a sequence of linear regressions before proceeding to the classification of observations to classes by comparing group centroids (Hastie et al. 2017). This assumption meant that the problem related to the separation of data with non-linear trends could be generalized as a sequence of polynomial regressions, assuming that the function that best describes the trend of the data is not a line, but a polynomial. The polynomial regression function (y = b_0_ + b_1_x_1_ + b_2_x_1_^2^ + b_2_x_1_^3^ + ......,b_n_x_1_^n^) consists of a linear model modified by adding polynomial parameters to the generic linear function, resulting in a “smoothing” of the line which better adapts the resulting curve to the actual distribution observed in the data (Gergonne, 1974). Flexible discriminant analysis (FDA) calculates a series of polynomial parameters up to the one that best fits the data, thus forming a new and more flexible separation decision boundary, increasing the performance of the model compared to a standard LDA, and lowering the margin of error rate while finding an optimal classification of the data (Hastie et al. 1994, Hastie et al. 2007). An alternative to polynomials is multivariate adaptive regression splines (MARS), an approach which captures the nonlinear relationships observed in the data by assessing cutpoints (*knots*). More in detail, the MARS algorithm first looks for the single point across the range of variable X values where two different linear relationships between variable Y and variable X achieve the smallest error. The result is a hinge function given in the form of *h(x−a)* where *a* is the cutpoint value. This procedure continues iteratively until many *knots* are found. Once the full set of knots has been identified, in order to prevent overfitting, a *pruning* process sequentially remove knots that do not contribute significantly to predictive accuracy, relying on an expected change in *R^2^* of less than 0.001. This calculation is automatically performed by the generalized cross-validation (GCV) procedure, which is a computational shortcut for these models that produces an approximate leave-one-out cross-validation (LOOCV) error metric (Golub et al. 1979).

***Random forest***

Random forest (Breiman et al. 2001) consists of a large number of individual decision trees that operate as an ensemble in order to increase the overall result (prediction). Each individual tree in the random forest classification output a class prediction and the class with the most votes becomes the model’s prediction. Each tree is grown following these procedures: 1) random sampling from the input data with replacement; 2) with M input variables, a number m<M is specified such that at each node, m variables are selected at random out of M and the best split on this m subset is used to split the node, helding the value of m constant the forest is growing; 3) each tree is grown to the largest extent possible without pruning. Variable choice at each node depends on a variety of measures of classification error, such as classification error rate (the maximum portion of observations that do not belong to the most common class) or Gini index (the sum across all classes of the products between class evenness and class error rate computed at each node) (Breiman et al. 2001). Moreover, when the training set for the current tree is drawn by sampling with replacement, about one-third of the cases are left out of the sample. This automatic procedure is the so called out-of-bag (OOB) and is used to get a running unbiased estimate of the classification error as trees are added to the forest (Breiman et al. 2001).

***Repeated k-fold cross-validation***

As stated above, both GCV and OOB produce an approximation of cross-validation, making it unnecessary to repeat the process. However, it has been established (Kim, 2009, Kuhn & Johnson 2013) that highly adaptive classifier and small/modestly-sized dataset problems require an integration with a repeated k-fold cross-validation. Indeed, the simple k-fold cross-validation procedure is a standard method for estimating the performance of a machine learning algorithm, but it may result in a noisy estimate of the model performance: each time the procedure is run, a different split of the dataset into k-folds is implemented, and so the distribution of performance scores can be different, resulting in a different mean estimate of model performance. With repeated k-fold cross-validation the estimated performance of a machine learning model is improved. This procedure simply involves the repetition of the k-fold cross-validation procedure multiple times and report the mean result across all folds and all repeats performed on the same dataset split into different folds. This gives a more robust model assessment score than performing cross-validation only once (Kim, 2009, Kuhn & Johnson 2013).


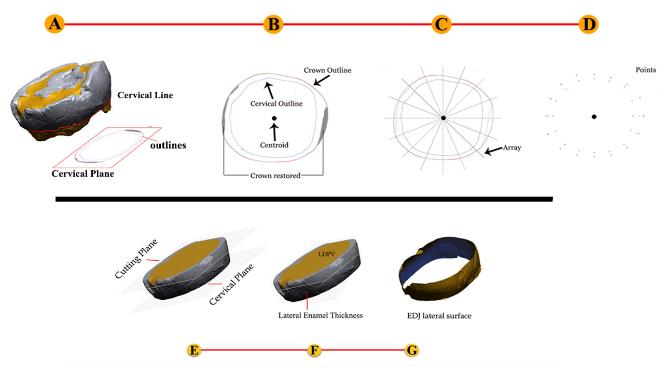
**Figure S1**. Sequence of lateral enamel thickness analysis.


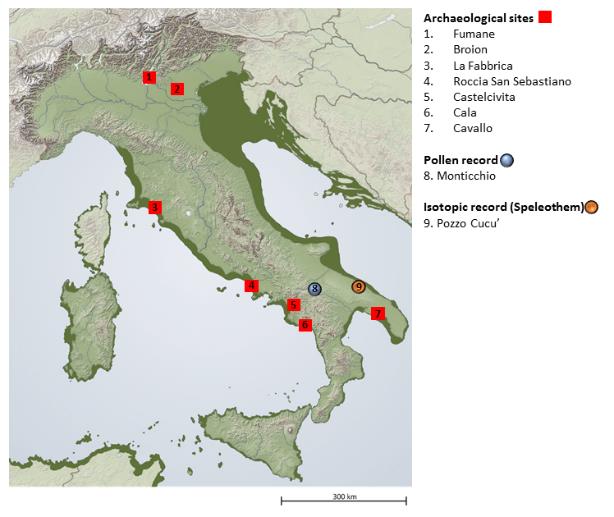


**Figure S2.** Paleogeographic map of Italy. Presence of Uluzzian groups at around 43-45 ka cal BP and on the Tyrrenian coast at around 40 ± 1.6 ka Optically stimulated luminescence (OSL).


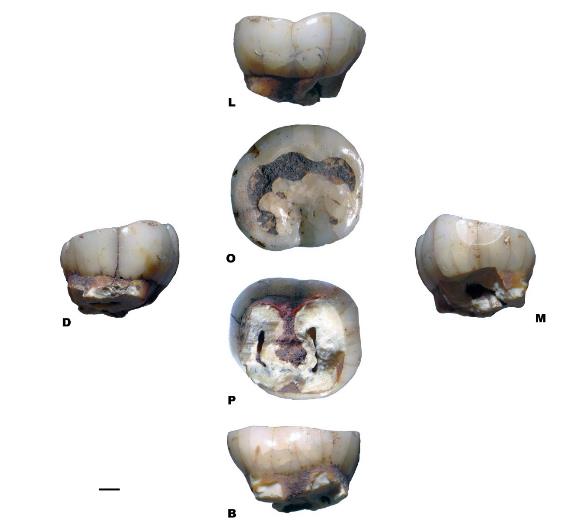


**Figure S3.** RSS2 (lower left second deciduous molar). Six views comprehensive of the root after sampling in the centre (P). O = Occlusal; P = periapical; B = buccal; D = distal; L = lingual; M = mesial. Scale bar: 2 mm


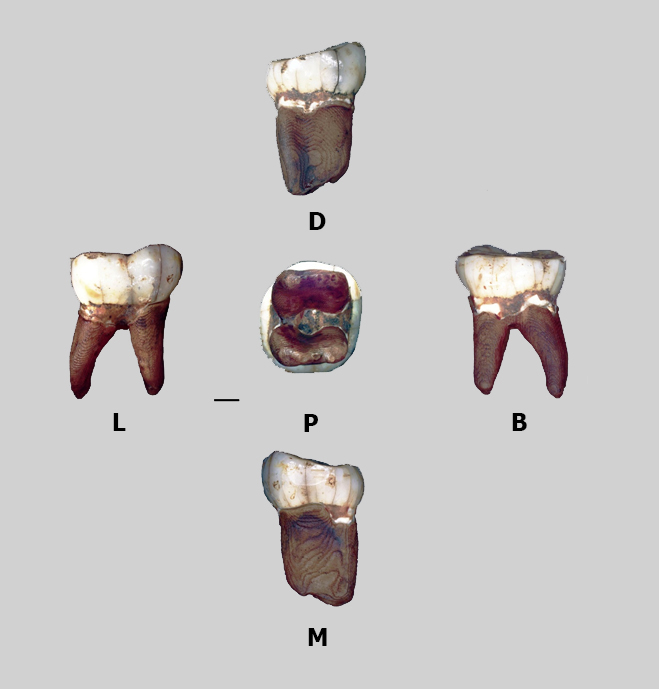


**Figure S4.** RSS2 (lower left second deciduous molar). Five comprehensive views of the tooth after restoration. P = periapical; B = buccal; D = distal; L = lingual; M = mesial. Scale bar: 2 mm

**Table S1.** Stratigraphic sequence correlation.

| Unit | Sub-unit | Cultural attribution | E14-E15 trench | F14 trench | E 16 trench |
| --- | --- | --- | --- | --- | --- |
| Unit 1 | C-Ca | Recent Gravettian | t1-t4 | t1-t4 | t1-t4 |
|  | Cb | Gravettian with Noailles burins | t5-t10 | t5-t10 | t5-t10 |
|  | Cc | Early Gravettian | t11-t17 | t11-t17 | t11-t15 |
| Unit 2 | Cd | Initial Gravettian | t18-t21 |  |  |
|  | Ce | Protoaurignacian with Dufour bladelets | t22-t26 |  |  |
| Unit 3 | Cf | Uluzzian | t27-t28 | t18-t20 | t16-t18 |
|  | Cg | Mousterian | t29-t34 | t21-t39 | t19-t25 (excavation ongoing) |

**Table S2.** Permutational multivariate analysis of variance test (with Bonferroni correction) of the crown outlines between recent *Homo sapiens* (RHS), Upper Paleolithic *Homo sapiens* (UPHS) and Neanderthals (N).

|  | RHS | N |
| --- | --- | --- |
| N | 0.003 |  |
| UPHS | 0.08 | 0.07 |

**Table S3.** Permutational multivariate analysis of variance test (with Bonferroni correction) of the cervical outlines between Recent *Homo sapiens* (RHS), Upper Paleolithic *Homo sapiens* (UPHS) and Neanderthals (N).

|  | RHS | N |
| --- | --- | --- |
| N | 0.003 |  |
| UPHS | 0.08 | 0.003 |

**Table S4.** Shapiro-Wilcoxon normality test for crown outlines

|  | PC1 | PC2 | PC3 | PC4 | PC5 | PC6 | PC7 | PC8 |
| --- | --- | --- | --- | --- | --- | --- | --- | --- |
| MH | 0.04 | 0.7 | 0.04 | 0.4 | 0.5 | 0.9 | 0.1 | 0.006 |
| N | 0.03 | 0.2 | 0.8 | 0.9 | 0.8 | 0.3 | 0.6 | 0.7 |

Abbreviations: PC = principal component; MH = modern humans; N = Neanderthals

**Table S5.** Fligner-Killeen homogeneity of variance test for crown outlines

|  | PC1 | PC2 | PC3 | PC4 | PC5 | PC6 | PC7 | PC8 |
| --- | --- | --- | --- | --- | --- | --- | --- | --- |
| *p*-values | 0.6 | 0.06 | 0.2 | 0.2 | 0.01 | 0.9 | 0.5 | 0.07 |

Abbreviations: PC = principal component.

**Table S6.** Shapiro-Wilcoxon normality test for cervical outlines

|  | PC1 | PC2 | PC3 | PC4 | PC5 | PC6 | PC7 | PC8 |
| --- | --- | --- | --- | --- | --- | --- | --- | --- |
| MH | 0.3 | 0.8 | 0.8 | 0.5 | 0.3 | 0.07 | 0.9 | 0.5 |
| N | 0.8 | 0.9 | 0.9 | 0.9 | 0.2 | 0.9 | 0.4 | 0.5 |

Abbreviations: PC = principal component; MH = modern humans; N = Neanderthals

**Table S7.** Fligner-Killeen homogeneity of variance test for cervical outlines

|  | PC1 | PC2 | PC3 | PC4 | PC5 | PC6 | PC7 | PC8 |
| --- | --- | --- | --- | --- | --- | --- | --- | --- |
| *p*-values | 0.1 | 0.9 | 0.3 | 0.9 | 0.02 | 0.4 | 0.4 | 0.6 |

Abbreviations: PC = principal component.

**Table S8.** Posterior Probabilities obtained from supervised learning models on crown and cervix data.

| Crown Posterior Probability | |  |  |  | Cervix Posterior Probability | | |  |
| --- | --- | --- | --- | --- | --- | --- | --- | --- |
|  |  | MH | N |  |  |  | MH | N |
|  | RSS1_Original | 1,35E+06 | 0.9999986504 |  | FDA | RSS1_Original | 0.01016926 | 0.989830741 |
| FDA | RSS1_Restored | 4,27E-01 | 0.9999995731 |  |  | RSS2 | 0.9954530 | 0.004546943 |
|  | RSS2 | 1,00E+06 | 0.0002366003 |  | MARS | RSS1_Original | 2,22E-10 | 1.00000000 |
|  | RSS1_Original | 0.0003339172 | 0.99966608 |  |  | RSS2 | 9,29E+05 | 0.07076497 |
| MARS | RSS1_Restored | 0.0001677156 | 0.99983228 |  | RF | RSS1_Original | 0.322 | 0.678 |
|  | RSS2 | 0.9938165697 | 0.00618343 |  |  | RSS2 | 0.922 | 0.078 |
|  | RSS1_Original | 0.178 | 0.822 |  |  |  |  |  |
| RF | RSS1_Restored | 0.188 | 0.812 |  |  |  |  |  |
|  | RSS2 | 0.794 | 0.206 |  |  |  |  |  |

Abbreviations: FDA= Flexible Discriminant Analysis; MARS= Multivariate adaptive regression splines; RF= Random Forest; MH= Modern Humans; N=Neanderthals.

**Table S9.** Roccia San Sebastiano 1 (RSS1) lateral enamel thickness measurements of individuals belonging to the comparative sample.

| Specimen/group | *n* | Enamel volume | LDPV  mean(SD) | Range | LEDJ | 3D LAET Mean(SD) | 3D LRET Mean(SD) |
| --- | --- | --- | --- | --- | --- | --- | --- |
| RSS1 |  | 23.5 | 127.37 |  | 69.06 | 0.34 | 6.76 |
| MH^a^ | 18 |  | 138.20 (19.49) | 103.70-168.50 |  | 0.41 (0.05) | 7.91 (0.85) |
| N^a^ | 10 |  | 183.70 (28.22) | 132.80-227.20 |  | 0.37 (0.07) | 6.54 (1.17) |

Abbreviations: LDPV = lateral dentine and pulp volume; LEDJ = enamel-dentine junction lateral surface area; 3DLAET = 3D lateral average enamel thickness; 3DLRET = 3D lateral relative enamel thickness; MH = modern humans; N = Neanderthals.

^a^ Data source: Benazzi et al. 2011 (Table 3).

**Table S10.** Roccia San Sebastiano 2 (RSS2) lateral enamel thickness measurements of individuals belonging to the comparative sample.

| Specimen | N | Lateral Enamel Volume |  | LDPV  mean(SD) | Range | LEDJ | 3D LAET  mean(SD) | 3D LRET  mean(SD) |
| --- | --- | --- | --- | --- | --- | --- | --- | --- |
| RSS2 |  | 48.41 |  | 153.94 |  | 78.57 | 0.62 | 11.50 |
| MH^a^ | 18 |  |  | 138.20 (19.49) | 103.70-168.50 |  | 0.41 (0.05) | 7.91 (0.85) |
| N^a^ | 10 |  |  | 183.70 (28.22) | 132.80-227.20 |  | 0.37 (0.07) | 6.54 (1.17) |

Abbreviations: LDPV = lateral dentine and pulp volume; LEDJ = enamel-dentine junction lateral surface area; 3DLAET = 3D lateral average enamel thickness; 3DLRET = 3D lateral relative enamel thickness; MH = modern humans; N = Neanderthals.

^a^Data source: Benazzi et al. 2011 (Table 3).

| Library ID^a^ | Sample | Number of molecules | Number of spike-in oligo | Shotgun data | | | | Enriched for human mtDNA | | | |
| --- | --- | --- | --- | --- | --- | --- | --- | --- | --- | --- | --- |
|  |  |  |  | Raw number of sequences | Unique sequences L>35, MQ>25 | 5’ C to T (95% CI) | 3’ C to T (95% CI) | Raw number of sequences | Unique sequences L>30 | 5’ C to T (95% CI) | 3’ C to T (95% CI) |
| A19717 | ~5 mg | 8.69E+07 | 1.06E+06 | 1,199,759 | 655 | 0.0  (0.0-2.7) | 0.0  (0.0-2.6) | 397,953 | 499 | 5.7  (1.9-12.9) | 3.9  (1.1-9.6) |
| A19714 | <1 mg | 1.46E+08 | 1.11E+06 | 922,296 | 35,330 | 0.8  (0.5-1.0) | 0.4  (0.2-0.5) | 368,746 | 11,352 | 1.5  (1.1-2.0) | 0.9  (0.5-1.2) |
| A19724 | ENC | 8.94E+07 | 9.59E+05 | 111,710 | 1,161 | 2.5  (0.7-4.7) | 0.8  (0.1-2.8) | 78,098 | 2,190 | 2.0  (0.8-3.4) | 1.6  (0.7-3.1) |
| A19631 | LNC | 7.62E+07 | 1.37E+06 | 86,101 | 8 | 0.0  (0.0-84.2) | 0.0  (0.0-97.5) | 62,081 | 6 | NA  (N/A) | 0.0  (0.0-84.2) |
| A19715 | LNC | 5.34E+07 | 7.69E+05 | 90,218 | 11 | 0.0  (0.0-70.8) | 0.0  (0.0-60.2) | 77,605 | 72 | 0.0  (0.0-18.5) | 7.7  (0.0-24.7) |

**Table S11.** Results of the DNA analysis of RSS1.

Abbreviations: ENC = extraction negative control; LNC = library preparation negative control.

^a^ Library A19717 was generated after placing the sample powder into a fresh tube; library A19714 after adding lysis buffer to the left-over powder in the original tube. Fragments originating from shotgun data were filtered for length (L) of at least 35 bp and mapping quality (MQ) of at least 25, and were only retained if they fell within areas of unique mappability (Map35_100% from Prüfer et al. 2014). After enrichment for human mtDNA, only mapped fragments longer than 30 bp were considered. Confidence intervals (CI) for the estimation of damage patterns (cytosine to thymine substitutions; C to T) are computed from a binomial distribution.

**Table S12**. Roccia San Sebastiano 1 (RSS1) buccolingual crown diameters (in mm) of the comparative sample.

|  | *n* | Mean  (SD) |
| --- | --- | --- |
| RSS1 |  | 9.03 |
| N^a^ | 34 | 9.40 (0.5) |
| Ehs^a^ | 8 | 9.80 (0.6) |
| RHS^a^ | 57 | 8.30 (0.6) |

Abbreviations: EHS = early *Homo sapiens*; RHS = recent *Homo sapiens*; N = Neanderthals; RSS1 = Roccia San Sebastiano 1

^a^Data source: Hershkovitz et al. 2011 (Table 4).

**Table S13.** Roccia San Sebastiano 2 (RSS2) buccolingual crown diameters (in mm) of the comparative sample.

|  | *n* | Mean  (SD) |
| --- | --- | --- |
| RSS2 |  | 9.16 |
| N^a^ | 34 | 9.40 (0.5) |
| Ehs^a^ | 8 | 9.80 (0.6) |
| RHS^a^ | 57 | 8.30 (0.6) |

Abbreviations: EHS = early *Homo sapiens*; RHS = recent *Homo sapiens*; N = Neanderthals; RSS2 = Roccia San Sebastiano 2

^a^Data source: Hershkovitz et al. 2011 (Table 4).

**References**

Aiello, G., Barra, D., Collina, C., Piperno, M., Guidi, A., Stanislao, C., … Donadio, C. (2018). Geomorphological and paleoenvironmental evolution in the prehistoric framework of the coastland of Mondragone, southern Italy. Quaternary International, 493, 70–85. https://doi.org/10.1016/j.quaint.2018.06.041

Allen, J. R. M., Brandt, U., Brauer, A., Hubberten, H. W., Huntley, B., Keller, J., … Zolitschka, B. (1999). Rapid environmental changes in southern Europe during the last glacial period. Nature, 400(6746), 740–743. https://doi.org/10.1038/23432

Allen, J. R. M., Watts, W. A., & Huntley, B. (2000). Weichselian palynostratigraphy, palaeovegetation and palaeoenvironment; the record from Lago Grande di Monticchio, southern Italy. Quaternary International, 73–74, 91–110. https://doi.org/10.1016/S1040-6182(00)00067-7

Belluomini, C., Calderoni, G., Collina, C., Fedi, F., Fiore, I., Gallotti, R., Pennacchioni, M., Piperno, M., Santangelo, N., Santo, A., Tagliacozzo, A. Tagliacozzo, A. (2007). La grotta di Roccia San Sebastiano (Mondragone, Caserta). Atti Della XL Riunione Scientifica “Strategia Di Insediamento Tra Lazio e Campania in Età Preistorica e Protostorica”, IIPP, 319–331.

Benazzi, S. et al. (2011). Comparison of dental measurement systems for taxonomic assignment of Neanderthal and modern human lower second deciduous molars, J. Hum. Evol. 61, 320-326 https://doi.org/10.1016/j.jhevol.2011.04.008.

Benazzi, S., Panetta, D., Fornai, C., Toussaint, M., Gruppioni, G., Hublin, J.J. (2014a). Technical Note: Guidelines for the digital computation of 2D and 3D enamelthickness in hominoid teeth. Am. J. Phys. Anthropol. 153, 305e313

Breiman, L. (2001). Random forests. Machine learning, 45, 5-32

Briggs, A.W. et al. (2007). Patterns of damage in genomic DNA sequences from a Neandertal. Proc. Natl. Acad. Sci. U. S. A. 104, 14616–14621

Brock, F., Ramsey, C. B., & Higham, T. (2007). Quality assurance of ultrafiltered bone dating. Radiocarbon, 49, 187–192. https://doi.org/10.1017/S0033822200042107

Collina, C., Benazzi, S., Marciani, G., Oxilia, G., Piperno, M., Repola, M. (2020). La Grotta paleolitica di Roccia San Sebastiano (Mondragone, CE). Nuovi dati e valorizzazione del patrimonio. In M. Carcaiso, A., Musella (Ed.), Patrimonio di conoscenza 2019. (pp. 11–24). Museo civico archeologico Biagio Greco.

Collina, C., Fiore, I., Gallotti, R., Pennacchioni, M., Piperno, M., Salvadei, L., T., & A. (2008). Il gravettiano di Roccia san Sebastiano (Mondragone, Caserta). In Il Tardiglaciale in Italia – Lavori in Corso (pp. 133–143).

Collina, C., Gallotti, R. (2007). L’industria litica di Grotta di Roccia San Sebastiano (Mondragone, Caserta). Risultati dello studio tecnologico. In XL Riunione Scientifica IIPP (pp. 331–347). Firenze.

Collina, C., Piperno, M. (2011). 40,000 anni di Preistoria a Mondragone. Nuove scoperte a Roccia San Sebastiano, Comune di Mondragone, Assessorato ai Beni e alle Attività Culturali, Grafica Reventino, Decollatura (CZ).

Collina, C., Piperno, M. (2018). La Grotta di Roccia San Sebastiano, Nuovi dati sulla sequenza paleolitica. In Patrimonio di conoscenza. Le terre del Massico, Museo civico archeologico Biagio Greco (pp. 14–21).

Columbu, A., Chiarini, V., Spötl, C., Benazzi, S., Hellstrom, J., Cheng, H., & De Waele, J. (2020). Speleothem record attests to stable environmental conditions during Neanderthal–modern human turnover in southern Italy (Nature Ecology & Evolution, (2020), 4, 9, (1188-1195), 10.1038/s41559-020-1243-1). Nature Ecology and Evolution, 4, 1188–1195. https://doi.org/10.1038/s41559-020-1267-6

Fletcher, W. J., Sánchez Goñi, M. F., Allen, J. R. M., Cheddadi, R., Combourieu-Nebout, N., Huntley, B., … Tzedakis, P. C. (2010). Millennial-scale variability during the last glacial in vegetation records from Europe. Quaternary Science Reviews, 29(21–22), 2839–2864. https://doi.org/10.1016/j.quascirev.2009.11.015

Gansauge, M.-T., Aximu-Petri, A., Nagel, S., Meyer, M. (2020). Manual and automated preparation of single-stranded DNA libraries for the sequencing of DNA from ancient biological remains and other sources of highly degraded DNA. Nat. Protoc. 15, 2279–2300

Gergonne, J.D. (1974). The application of the method of least squares to the interpolation of sequences. Hist. Math. 1, 439-447

Giaccio, B., Hajdas, I., Isaia, R., Deino, A., & Nomade, S. (2017). High-precision 14C and 40Ar/39 Ar dating of the Campanian Ignimbrite (Y-5) reconciles the time-scales of climatic-cultural processes at 40 ka. Scientific Reports, 7(March), 1–10. https://doi.org/10.1038/srep45940

Glocke, I. & Meyer, M. (2017). Extending the spectrum of DNA sequences retrieved from ancient bones and teeth. Genome Res. 27, 1230–1237

Golub, G. H., Heath, M., & Wahba, G. (1979). Generalized cross-validation as a method for choosing a good ridge parameter. Technometrics, 21, 215-223

Hastie, T., Tibshirani, R., and Buja, A. (1994). Flexible discriminant analysis by optimal scoring. J. Am. Stat. Assoc. 89, 1255-1270

Hastie, T., Tibshirani, R., and Friedman, J. (2017). The elements of statistical learning: data mining, inference, and prediction. Springer Science & Business Media,

Hershkovitz, I. et al. (2011). Middle pleistocene dental remains from Qesem Cave (Israel). Am. J. Phys. Anthropol. 144, 575–592

Kim, J. H. (2009). Estimating classification error rate: Repeated cross-validation, repeated hold-out and bootstrap. CSDA, 53, 3735-3745

Kono, R. T. (2004). Molar enamel thickness and distribution patterns in extant great apes and humans: New insights based on a 3-dimensional whole crown perspective. Anthropological Science. https://doi.org/10.1537/ase.03106

Korlević, P., Talamo, S., Meyer, M. (2018). A combined method for DNA analysis and radiocarbon dating from a single sample. Sci. Rep. 8, 4127

Kuhn, M., & Johnson, K. (2013). Applied predictive modeling. New York, Springer, 26, 1-16

Li, H., Durbin, R. (2009). Fast and accurate short read alignment with Burrows–Wheeler transform. Bioinformatics. 25, 1754–1760

Lowe, J., Barton, N., Blockley, S., Ramsey, C. B., Cullen, V. L., Davies, W., … Tzedakis, P. C. (2012). Volcanic ash layers illuminate the resilience of Neanderthals and early modern humans to natural hazards. Proceedings of the National Academy of Sciences of the United States of America, 109(34), 13532–13537. https://doi.org/10.1073/pnas.1204579109

Marciani, G., Ronchitelli, A., Arrighi, S., Badino, F., Bortolini, E., Boscato, P., … Benazzi, S. (2020). Lithic techno-complexes in Italy from 50 to 39 thousand years BP: An overview of lithic technological changes across the Middle-Upper Palaeolithic boundary. Quaternary International, 551, 123–149. https://doi.org/https://doi.org/10.1016/j.quaint.2019.11.005

Margari, V., Gibbard, P. L., Bryant, C. L., & Tzedakis, P. C. (2009). Character of vegetational and environmental changes in southern Europe during the last glacial period; evidence from Lesvos Island, Greece. Quaternary Science Reviews, 28(13–14), 1317–1339. https://doi.org/10.1016/j.quascirev.2009.01.008

Maricic, T., Whitten, M., Pääbo, S. (2010). Multiplexed DNA Sequence Capture of Mitochondrial Genomes Using PCR Products. PLoS One 5, 1–5

Meyer, M. et al. (2012). A High-Coverage Genome Sequence from an Archaic Denisovan Individual. Science. 338, 222–226

Nowaczewska, W. et al. (2021). New hominin teeth from Stajnia Cave, Poland. J. Hum. Evol. 151, 102929 - 102929

Prüfer, K. et al. (2014). The complete genome sequence of a Neanderthal from the Altai Mountains. Nature. 505, 43–49

Renaud, F., Bournaud, F., Kraljic, K., Duc, P.A., (2017). Starbursts triggered by intergalactic tides andinterstellar compressive turbulence. Mon. Not. R. Astron. Soc. 442, L33–L37 https://doi.org/10.1093/mnrasl/slu050.

Rohland, N. et al. (2018). Extraction of highly degraded DNA from ancient bones, teeth and sediments for high-throughput sequencing. Nat. Protoc. 13, 2447–2461 https://doi.org/10.1038/s41596-018-0050-5

Slon, V. et al. (2017). Neandertal and Denisovan DNA from Pleistocene sediments. Science 356, 605–608

Vazzana, A. et al. (2018). A multianalytic investigation of weapon-related injuries in a Late Antiquity necropolis, Mutina, Italy. J. Archaeol. Sci. Reports. 17, 550–559

Wacker, L. et al. (2010). Micadas: Routine and high-precision radiocarbon dating. Radiocarbon 52, 252–262
